# What Makes a Difference in Exercise-Induced Bronchoconstriction: An 8 Year Retrospective Analysis

**DOI:** 10.1371/journal.pone.0087155

**Published:** 2014-01-30

**Authors:** Han-Ki Park, Jae-Woo Jung, Sang-Heon Cho, Kyung-Up Min, Hye-Ryun Kang

**Affiliations:** 1 Department of Internal Medicine, Seoul National University College of Medicine, Seoul, Korea; 2 Institute of Allergy and Clinical Immunology, Seoul National University Medical Research Center, Seoul, Korea; 3 Department of Internal Medicine, Chung-Ang University College of Medicine, Seoul, Korea; University of Liverpool, United Kingdom

## Abstract

**Background:**

Exercise-induced bronchoconstriction (EIB) was recently classified into EIB alone and EIB with asthma, based on the presence of concurrent asthma.

**Objective:**

Differences between EIB alone and EIB with asthma have not been fully described.

**Methods:**

We retrospectively reviewed who visited an allergy clinic for respiratory symptoms after exercise and underwent exercise bronchial provocation testing. More than a 15% decrease of forced expiratory volume in 1 second (FEV1) from baseline to the end of a 6 min free-running challenge test was interpreted as positive EIB.

**Results:**

EIB was observed in 66.9% of the study subjects (89/133). EIB-positive subjects showed higher positivity to methacholine provocation testing (61.4% vs. 18.9%, p<0.001) compared with EIB-negative subjects. In addition, sputum eosinophilia was more frequently observed in EIB-positive subjects than in EIB-negative subjects (56% vs. 23.5%, p = 0.037). The temperature and relative humidity on exercise test day were significantly related with the EIB-positive rate. Positive EIB status was correlated with both temperature (p = 0.001) and relative humidity (p = 0.038) in the methacholine-negative EIB group while such a correlation was not observed in the methacholine-positive EIB group. In the methacholine-positive EIB group the time to reach a 15% decrease in FEV1 during exercise was significantly shorter than that in the methacholine-negative EIB group (3.2±0.7 min vs. 8.6±1.6 min, p = 0.004).

**Conclusions:**

EIB alone may be a distinct clinical entity from EIB with asthma. Conditions such as temperature and humidity should be considered when performing exercise tests, especially in subjects with EIB alone.

## Introduction

Exercise-induced bronchoconstriction (EIB) is defined as a transient narrowing of the lower airway following exercise regardless of the presence of asthma. The term *exercise-induced asthma* is not widely used currently because exercise is not an independent risk factor for asthma but a trigger of bronchoconstriction in underlying asthma. The term EIB reflects more accurately on the underlying pathophysiology because it includes patients with chronic asthma in which exercise triggers bronchoconstriction and patients with bronchoconstriction associated with exercise but without chronic asthma [1], [2]. EIB frequently accompanies chronic asthma and 40% to 90% of asthma patients exhibit EIB [3]–[5]. In addition to EIB in chronic asthma, EIB can develop in non-asthmatic general populations with prevalence ranging between 8% and 20% [2], [3].

Vigorous exercise is a well-known aggravating factor of asthma [1], [6] and EIB accompanying asthma can be an indicator of the state of asthma control [7], [8]. While EIB commonly occurs in asthmatics, it is also reported in individuals without other clinical asthma symptoms and EIB in elite athletes have been extensively investigated in the field of sports medicine. Many studies have reported risk factors for the development of EIB in non-asthmatic athletes, such as environmental conditions, type of exercise, pathogenesis, and treatments [9]–[11]. There are some reports suggesting that EIB in chronic asthmatics is distinct from EIB in athletes. For example, steroid responses, positivity in methacholine provocation tests, and inflammatory patterns in sputum are reported to be different between the two conditions [3], [9]. However, EIB without asthma in non-athlete adults is not widely investigated, especially adult.

In the present study, we analyzed several factors related with EIB positivity in exercise provocation testing, and compared EIB-only and EIB with asthma groups to determine if there were clinical differences between these two phenotypes.

## Methods

### 1. Ethical Consideration

Research in the planning and execution was followed the 2008 59th World Medical Association (WMA) General Assembly in Seoul, the latest revised compliance with the Declaration of Helsinki and Institutional Review Board (IRB) approved the study protocol by th ethics committee our hospital was conducted in accordance with. Our study method was retrospective chart review. So we could not obtain informd consent and ethics committee of Seoul National University Hospital admitted in the absence of informed consent.

### 2. Study Subjects

We retrospectively reviewed electronic medical records of patients who visited an allergy clinic for respiratory symptoms after exercise and underwent exercise bronchial provocation testing from January 2004 to December 2012. Patients older than 18 years of age presenting with respiratory symptoms while exercising who underwent exercise provocation testing were included in the study. Seven female patients, out of a total of 140, have been excluded for comparison by gender was not possible. One hundred seven patients underwent methacholine bronchial provocation testing, and the patients were divided into groups and analyzed based on the presence of methacholine hypersensitivity and positivity to exercise testing. All exercise tests were performed between 11∶00 a.m. and 12∶00 p.m. Temperature and humidity of the test day at 12∶00 p.m. in the Jongno district, where the outdoor exercise test was performed, were obtained from the data of the Korean Meteorological Administration (http://www.kma.go.kr) and were interpreted as the test conditions. We accessed the online open system of Korea Environment Corporation (http://www.airkorea.or.kr) and retrieved SO_2,_ CO_2,_ CO, O_3_, and fine dust (PM_10_) data to obtain test day air pollution levels.

In addition to the exercise challenge results, methacholine challenge test, skin prick test for common inhalant allergens, serum IgE levels measured by ImmunoCAP®, inflammatory nature of induced sputum, and paranasal sinus view results were also retrieved and included in the analysis.

### 3. Measurements

The exercise challenge comprised 6 minutes of outdoor free running. Medical records show that the subjects’ maximum heart rate reached 85% of the estimated maximum before the termination of exercise. Forced expiratory volume in 1 second (FEV1) was measured before the initiation of exercise and at 1, 3, 5, 10, 15, 20, 30, 45, and 60 min after exercise. Presence of EIB was identified if post-exercise FEV1 decreased by 15% or more from the pre-exercise value.

Airway hyper-responsiveness to methacholine was measured by using the method suggested by Chai et al. [12] Methacholine bronchial challenge was performed on another day within one week of the EIB free-running test. Methacholine hyper-responsiveness was defined as positive when the provocation methacholine concentration inducing a 20% reduction in FEV1 (PC_20_) was less than 16 mg/mL.

Sputum induction was performed to evaluate the inflammatory cells in sputum as previously reported. [13] Sputum eosinophilia was defined as eosinophils ≥3% of total inflammatory cells seen in the sputum sample.

Skin prick tests (Allergopharma®, Reinbeck, Germany) included tests for sensitivity to house dust mites (*Dermatophagoides pteronyssinus* and *D. farinae*), outdoor mold mixture, indoor mold mixture, cat fur, dog fur, cockroach, tree pollen mixture, grass mixture, and weed mixture. Atopy was defined by one or more positive skin prick test responses.

In study subjects, asthma medications were discontinued at least one week prior to above tests. The European Community of Coal and Steel reference equations were used for a percentage of predicted value for FEV1 and FVC.

### 4. Statistical Analysis

Statistical analysis was performed using SPSS for Windows version 17.0. Data are presented as means ± standard error or as percentages. Independent *t*-test was used for analysis of continuous variables. Categorical variables are shown as frequencies or percentages and were analyzed by chi-square tests. A *p* value of <0.05 was considered statistically significant.

## Results

### 1. Demographic and Clinical Characteristics of the Study Subjects

One hundred thirty-three patients were included and 89 were EIB positive following exercise challenge testing; 44 were EIB negative. The mean age of the patients was 22.8 years; 120 patients, 90.2%, of the patients enrolled were under 30 years old. 84.7% of the patients were atopic. There were no differences between the groups with regard to smoking and history of asthma. There were no professional or elite athletes among the study subjects. Airway hyper-responsiveness to methacholine was detected in 46.7% of the study patients. Mean levels of air pollution indices, such as SO_2,_ CO_2,_ CO, O_3_, and fine dust (PM_10_), were within levels considered acceptable by the Korean government. (Table 1).

**Table 1:** Clinical characteristics of subjects.

	Total N = 133	EIB (+) N = 89	EIB (−) N = 44	P value
Age (yr)	22.8±0.5	22.8±0.6	22.7±0.8	0.912
Smoking status (none/total, %)	67.1	64.8	71.4	0.719
Temperature (°C)	12.1±1.0	9.9±1.2	16.4±1.8	**0.003**
Humidity (%)	47.3±1.3	44.9±1.4	52.1±2.4	**0.008**
**Atopy rate (%)**	84.7	88.9	77.4	0.157
**Serum IgE (mean, IU/mL)**	546.46±103.1	651.43±139.7	326.95±118.2	0.142
**Sinusitis (%)**	23.3	29.8	11.5	0.077
**Sputum analysis**				
Macrophage (%)	76.2±2.5	75.0±3.6	77.9±3.3	0.588
Neutrophil (%)	13.2±1.8	14.1±2.8	12.0±1.9	0.583
Eosinophil (%)	8.4±2.2	10.0±3.1	6.2±2.8	0.401
Eosinophilia rate (%)^a^	42.9	56.0	23.5	**0.037**
**Pulmonary function test**				
FEV1 (%)	90.7±1.4	87.8±1.7	96.3±2.0	**0.003**
FVC (%)	92.5±1.1	90.9±1.3	95.5±2.2	0.055
FEV1/FVC (%)	83.6±0.8	82.0±1.1	85.7±0.7	**0.004**
**Methacholine provocation test**				
Positive rate (%)^b^	46.7	61.4	18.9	**<0.001**
PC_20_ (mg/mL)	8.0±0.9	7.1±1.0	12.9±1.5	**0.020**
**Exercise provocation test**				
Decrement of FEV1 (%)	22.5±1.2	29.7±1.3	7.9±0.6	<0.001
**Air pollution**				
SO_2_	0.0070±0.0002	0.0070±0.0003	0.0069±0.0003	0.963
CO_2_	0.0357±0.0011	0.0357±0.0015	0.0357±0.0017	0.992
CO	0.64±0.028	0.67±0.04	0.58±0.03	0.102
O_3_	0.018±0.002	0.017±0.002	0.020±0.003	0.365
Fine dust (PM_10_)	51.6±2.1	49.77±2.24	55.15±4.21	0.264

Data are means ± SEM or percentages; EIB, exercise provocation test,

a eosinophilia ≥3%,

b positive when PC_20_>16 mg/mL

c air – environmental standards of Korea: SO_2_<0.05 ppm (24 h), CO <9 ppm (8 h), O_3_<0.06 ppm (8 h), PM_10_<100 µg/m^3^ (24 h).

### 2. Comparison of Clinical and Environmental Characteristics between EIB-positive and EIB-negative Groups

There were no differences between the positive and negative EIB groups with regard to atopy rate, serum total IgE levels, and sensitization rate to individual inhalant allergens except for the sensitization rate to outdoor molds, which was higher in the EIB-positive group (24.1% vs. 6.5%, *p* = 0.044) (Table 1).

Sputum examination revealed that the proportion of macrophage, neutrophil and eosinophil (10.0±3.1 vs. 6.2±2.8) was not different between the two groups. However, sputum eosinophilia was more frequently detected in the EIB-positive group (Table 1).

**Figure 1:**
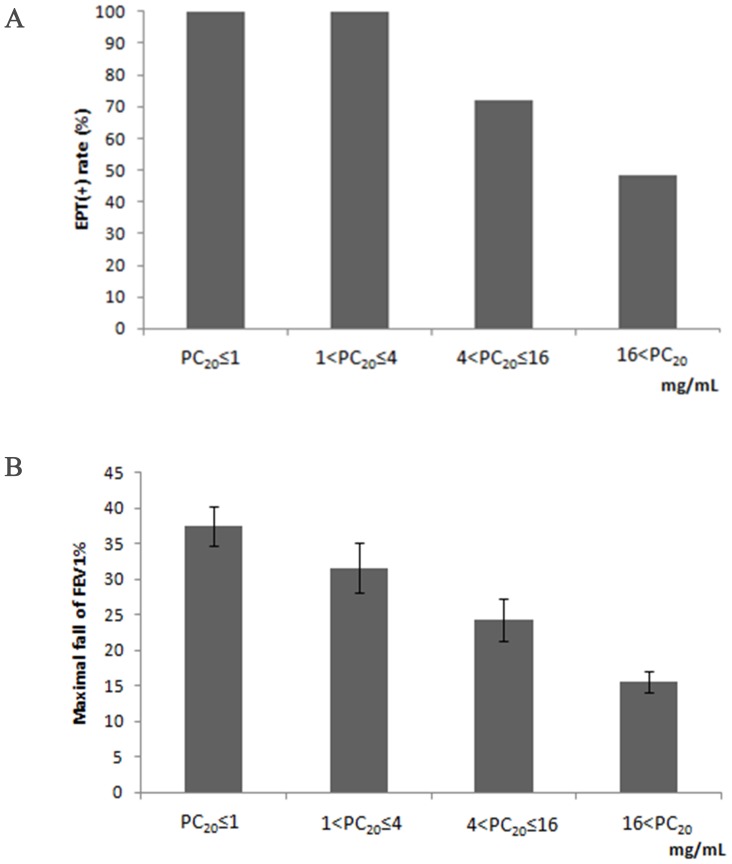
Positive rate and maximal fall of FEV1% of exercise provocation test according to PC_20_. (A) Positive EIB rate and (B) maximal decrease in FEV1.

With regard to pulmonary function parameters, baseline FEV1 was approximately 10% lower in the EIB-positive group (*p = *0.003). In addition, the EIB-positive group had a positive methacholine challenge rate three times higher than that of the EIB-negative group (61.4% vs. 18.9%, *p*<0.001). The exercise challenge positive rate was markedly higher in subjects who were more sensitive to methacholine provocation and all subjects with a PC_20_<4 mg/mL exhibited EIB on exercise challenge (Fig. 1A). The maximal decrease in FEV1 was also higher in subjects who were more sensitive to methacholine provocation (Fig. 1B).

**Figure 2:**
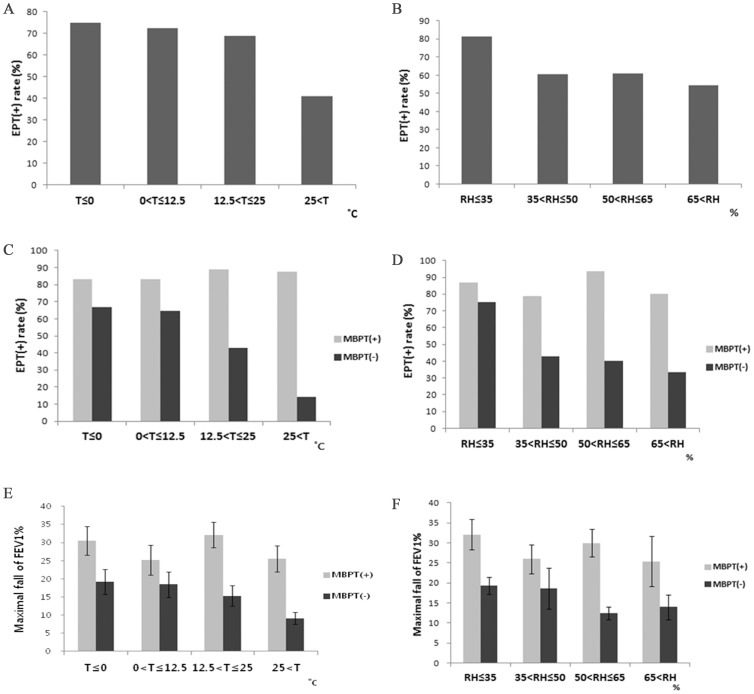
Positive rate and maximal fall of FEV1% at different air temperature and relative humidity levels. (A–D) Positive EIB rates and (E–F) maximal decrease in FEV1.

In the EIB-positive group, test day temperature and relative humidity levels were significantly lower than those experienced by the EIB-negative group (*p*<0.01). The differences in mean temperature and relative humidity were 6.5°C and 7.2%, respectively (Table 1). After categorizing air temperature into four segments, the EIB-positive rate was highest (approximately 75%) when the temperature was below 0°C (Fig. 2A). Similarly, the EIB-positive rate was highest (above 80%) when the relative humidity was below 35% (Fig. 2B). Air pollution indices (SO_2,_ CO_2,_ CO, O_3_, and fine dust) did not show any association with EIB positivity.

### 3. Comparison of Clinical Characteristics on the Basis of Presence of Hypersensitivity to Exercise and Methacholine

The study subjects were classified on the basis of exercise and methacholine challenge test results (Table 2). Among the 50 methacholine hyper-responsive patients, the exercise challenge positive rate was 86%. Pulmonary function parameters were relatively lower in EIB-positive patients than in EIB-negative patients, but the differences were not statistically significant. Patients hyper-responsive to both methacholine and exercise were more sensitive to the methacholine challenge and their mean PC_20_ was significantly lower than that in patients hyper-responsive to methacholine only (*p = *0.007). In methacholine challenge positive patients, clinical characteristics, such as atopy rate, serum IgE levels, eosinophilia rate, and baseline PFT, were similar irrespective of exercise hyper-responsiveness.

**Table 2:** Clinical characteristics of four groups based on the results of free-running provocation and methacholine provocation tests.

	MBPT (+)		MBPT (−)	
	EIB (+) N = 43	EIB (−) N = 7	EIB (+) N = 27	EIB (−) N = 30
Age (mean, yr)	21.7±0.6	20.6±0.6	24.1±1.5	23.6±1.1
Nonsmoker (None/total, %)	21/30 (70)	4/6 (66.7)	10/16 (62.5)	15/20 (75)
History of asthma (%)	24 (55.8)	2 (28.6)	9 (33.3)	11 (36.7)
Temperature (mean °C)*	12.0±1.9	11.0±4.7	7.4±2.2	17.8±2.1
Humidity (mean %)*	45.7±2.2	47.4±8.9	45.4±2.4	52.3±2.2
**Atopy rate (%)**	93.5	100	77.8	72
**Serum IgE (mean, IU/mL)**	759.7±149.3	185.3±50.5	233.4±53.8	375.1±151.5
**Sinusitis (%)**	29.6	0	35.3	15.8
**Sputum analysis**				
Macrophage (mean, %)	76.5±3.7	68.5±8.2	69.1±9.3	79.6±3.7
Neutrophil (mean, %)	13.7±3.5	18.7±4.1	15.8±5.6	10.4±2.1
Eosinophil (mean, %)	8.7±2.8	11.3±7.1	14.5±9.0	5.3±3.4
Eosinophilia rate	70.6	66.7	57.1	15.4
**Pulmonary function test**				
FVC%	89.6±1.7	93.86±4.3	93.0±1.8	95.86±2.5
FEV1%	83.5±2.2	93.14±4.3	94.7±2.1	97.03±2.3
FEV1/FVC%	79.2±1.4	84.29±1.3	86.6±1.4	86.08±0.8
**Methacholine provocation**				
PC_20_ (mg/mL)**	5.3±0.8	11.1±1.0		
**Exercise provocation test**				
Decrement of FEV1 (%)	32.6±1.7	6.93±1.1	24.6±2.1	7.40±0.7
**Air pollution**				
SO_2_	0.0072±0.0004	0.0076±0.0008	0.0071±0.0007	0.0070±0.0004
CO_2_	0.0375±0.0021	0.0324±0.0025	0.0354±0.0030	0.0367±0.0021
CO	0.68±0.06	0.47±0.06	0.68±0.35	0.62±0.23
O_3_	0.015±0.002	0.016±0.002	0.020±0.033	0.022±0.019
Fine dust	51.36±3.35	54.43±10.05	50.15±19.06	55.63±28.93

Data are means ± SEM or percentages, MBPT: methacholine bronchial provocation test; EIB: exercise provocation test.

* indicates *p*<0.05 between EIB(+) and EIB(−) patients among MBPT(−) patients.

** indicates *p*<0.05 between EIB(+) and EIB(−) patients among MBPT(+) patients.

Among the 57 methacholine non-responders, 27 patients (47.4%) exhibited EIB and pulmonary function parameters were not significantly different between EIB-positive patients and EIB-negative patients.

Among the four patient groups classified according to the presence of methacholine hypersensitivity and EIB, total IgE was remarkably elevated in methacholine-positive EIB subjects but atopy rate was not different.

**Figure 3:**
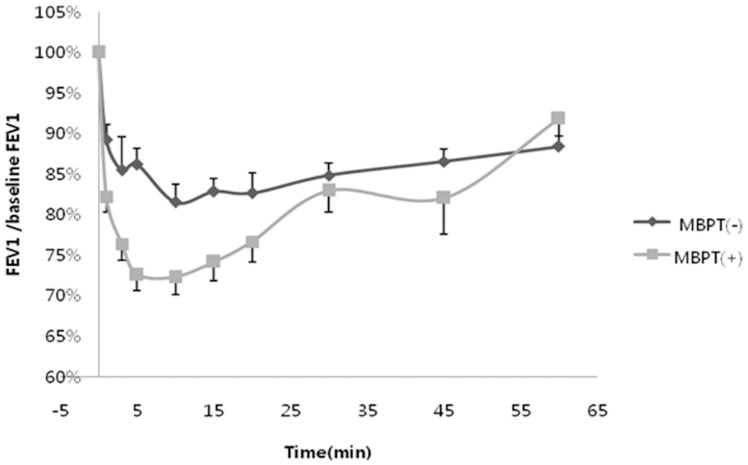
Decrement in FEV1 during exercise provocation test in patients with EIB based on methacholine reactivity. MBPT: methacholine bronchial provocation test.

Although patients with EIB exhibited relatively lower lung function, baseline pulmonary function parameters were not statistically different. However, methacholine-positive EIB patients showed a steeper slope of FEV1 decrease during exercise challenge testing compared with methacholine-negative EIB patients. In other words, airflow limitation development in methacholine-positive EIB patients was relatively more abrupt and severe compared with methacholine-negative EIB patients (Table 3 and Fig. 3).

**Table 3:** Time to reach ΔFEV1≥15% during free-running tests among patients with positive exercise provocation test results.

	EIB(+),MBPT(−)	EIB(+),MBPT(+)	*P* value
**Time to reach** Δ**FEV1≥15%**	8.6±1.6	3.2±0.7	0.004

### 4. Comparison of Environmental Conditions on the Basis of Presence of Hypersensitivity to Exercise and Methacholine

**Figure 4:**
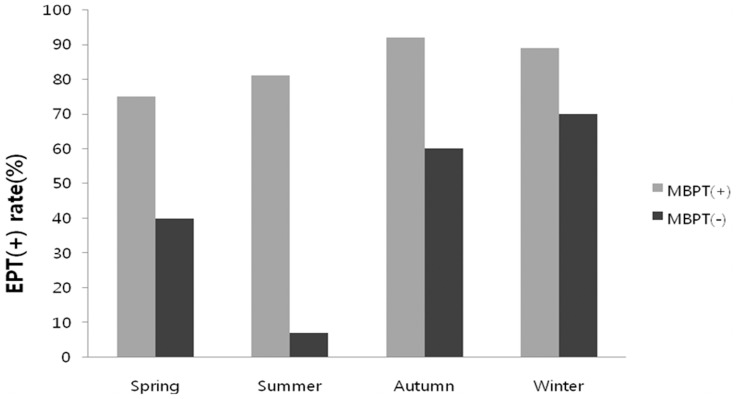
Positive EIB rate in free-running provocation tests based on season of testing. Spring: March to May, Summer: June to August, Autumn: September to November, Winter: December to February.

Test conditions, such as temperature and humidity, did not differ on the basis of EIB positivity in methacholine hyper-responsive patients, but those conditions were associated with EIB positivity in patients without methacholine hypersensitivity. Isolated EIB subjects had their test in colder conditions compared with all other subgroups. Similarly, the EIB-positive group underwent exercise testing on less humid conditions than those experienced by the EIB-negative group (*p = *0.038). When temperature and relative humidity data were divided into four categories, there was a linear relationship among the categories for EIB-positivity rate and maximal FEV1 decrease in methacholine non-responders, but this linearity was not observed in methacholine-sensitive patients (Fig. 2c–2f). In addition, seasonal differences were observed only in methacholine non-responders (Fig. 4).

**Figure 5:**
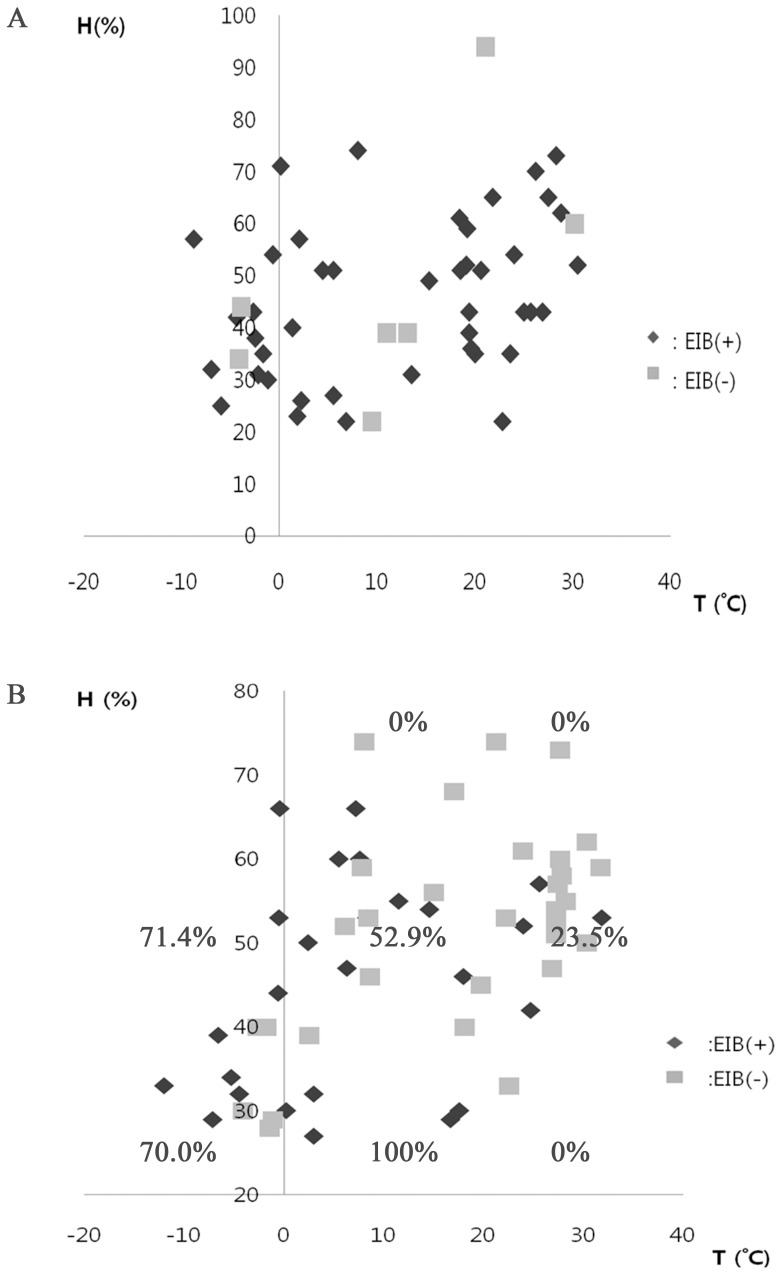
Distribution of patients by EIB status, air temperature, and relative humidity. (A) patients who showed positivity to methacholine challenge test and (B) patients who did not respond to methacholine challenge. Percent in graph is positive EIB rate.

With regard to the distribution of temperature and humidity on test days, test conditions were colder and less humid among methacholine non-responders while they were more evenly distributed in methacholine hyper-responsive patients (Fig. 5). After compartmentalizing test day conditions by temperature (<5°C, 5–20°C, >20°C) and humidity (<35%, 35–70%, >70%) in methacholine non-responders, these distribution patterns become more obvious. Within the 5°C to 20°C zone, there was a stepwise increase in EIB positivity as the relative humidity decreased (0%→52.9%→100%). Similarly, higher EIB positivity was observed in the lower temperature zone within the 35%∼70% relative humidity conditions (23.5%→52.9%→71.4%).

There was no relationship between the test day air pollution indices and either EIB positivity or methacholine hypersensitivity (Table 2).

### 5. Comparison of Airway Inflammation Through Sputum Exam on the Basis of Presence of Hypersensitivity to Exercise and Methacholine

Proportions of inflammatory cells (macrophage, neutrophil, and eosinophil) in sputum were not statistically different regardless of hypersensitivity to exercise. However, sputum eosinophilia was commonly observed in patients with either EIB or methacholine hypersensitivity and 57.1% of the isolated EIB patients showed sputum eosinophilia.

**Figure 6:**
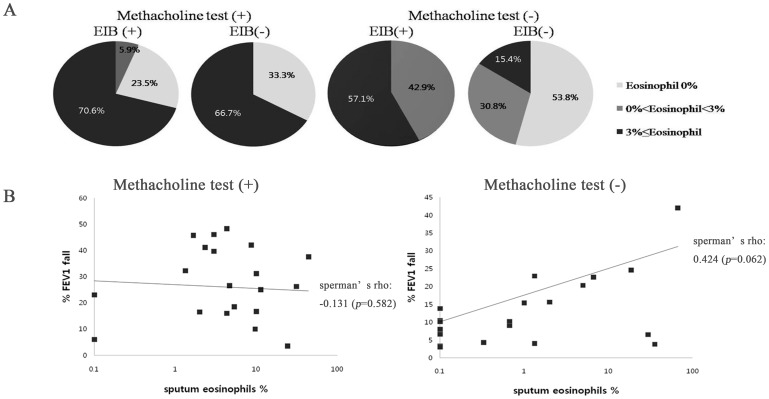
Eosinophilic inflammation at subgroups according to MBPT and EIB. (A) Sputum eosinophilia on the basis of hyper-responsiveness to methacholine and to exercise. (B–C) Relationship between sputum eosinophil presence and maximal fall in FEV1. EIB: exercise induced bronchoconstriction, MBPT: methacholine bronchial provocation test.

Sputum eosinophilia rates were higher in patients with EIB compared with patients without EIB regardless of methacholine hypersensitivity. Sputum eosinophilia was correlated with maximal reduction of FEV1 during exercise challenge testing in methacholine non-responders (Fig. 6). The more eosinophils present in sputum, the larger the maximal FEV1 fall (%) during exercise. However, this association was not observed in methacholine hypersensitive subjects.

## Discussion

The suggested pathophysiology of EIB consists of bronchoconstriction triggered by osmotic gradient or vasodilation as a result of re-warming the airway after exposure to cold and dry air [2], [9], [14]–[16]. Recently, the effects of inflammatory cells and their mediators on EIB have been investigated [1], [9], [16]–[18]. However, the exact pathophysiology of EIB has not been fully described.

There are several factors known to affect the development of EIB. In addition to host-related factors, environmental factors during exercise, such as cold and dry air, contributing to bronchospasm. [1], [19] Therefore, the method of examination can affect the result of an exercise challenge. [19].

In this study, baseline FEV1 and FEV1/FVC ratio, PC_20_, and sputum eosinophilia rate were different depending on the presence of EIB. It has been suggested that lower baseline FEV1 and PC_20_ levels along with more frequent sputum eosinophilia may reflect a less controlled state of asthma in asthmatics with EIB [7]. Atopy and upper airway diseases are also known to influence EIB [20]–[22]. Although higher sensitization to house dust mites has been reported [21], a significant difference in total IgE, atopy rate, and house dust mite sensitization rate was not observed in our study. Instead, we observed an increased sensitization rate to outdoor molds in EIB-positive patients.

Generally, maximal reduction of FEV1 occurs 5–10 min after exercise. Exercise itself is an indirect stimulus for the development of bronchoconstriction because of its association with airway dehydration. In contrast, methacholine directly constricts bronchi via the action of M3 receptors [23], [24]. Bronchoconstriction mechanisms of these two stimuli are different. However, hypersensitive responses to these two stimuli are reported to be closely related [23]. In the present study, the more sensitive to cholinergic stimuli the airway is, the more frequent the occurrence of EIB. For example, all patients with PC_20_<4 mg/mL exhibited bronchoconstriction triggered by exercise under every external condition observed. These findings indicate the susceptibility of uncontrolled asthmatics to exercise and the need to treat the underlying asthma when asthmatic patients experience symptoms during exercise.

Along with such patient factors, test day environmental factors had significant associations with the occurrence of EIB in our study. Temperature and humidity are two well-known factors reported to affect development of EIB [25]–[27]. Winter-sports athletes are often exposed to cold and dry air and are reported to have high frequency of occurrence of EIB [7], [28], [29]. Moreover, the sensitivity of the exercise challenge test has been reported to be lower during the summer compared with other seasons [30], [31]. Some previous studies have reported that humidity is more important than temperature in the development of EIB [32], [33]. In the present study, although both temperature and humidity seem to affect EIB positivity, humidity decrease in the same temperature zone showed a steeper increase of the EIB positive rate compared with the temperature decrease in the same humidity zone.

The most interesting finding in our study is that the association of EIB and temperature or humidity was observed only in isolated EIB patients. These negative associations between EIB positivity and temperature or humidity were not observed in methacholine hypersensitive patients; their EIB positivity was only affected by their hypersensitivity to methacholine. This result suggests that EIB alone and EIB with asthma are distinct disease entities with different pathomechanisms.

Ground level ozone and other air pollutants can trigger worsening of asthma symptoms and air pollution including ozone levels is a risk factor for EIB [27], [34], [35]. Although some previous studies have reported that air pollution had an effect on respiratory diseases only in high concentrations, repeated exposure to low concentration ozone can enhance responses to inhaled allergens in patients with preexisting airway diseases [36]. In the present study, association between the concentration of air pollutants and EIB positivity was not observed. However, considering the very low concentrations of air pollutants in the air on the test days in our study, the possibility of air pollutants affecting EIB positivity cannot be excluded. Recent studies on the effect of age on EIB show that prevalence of EIB in children is higher, children with EIB recover a little faster, and bronchoconstriction can occur during exercise in children [37]. Most of the subjects in this study were young adults over the age of 18 years and the difference due to age was not observed.

The pathogenesis of EIB is still poorly understood and many studies have suggested a non-inflammatory basis of EIB related to thermal fluxes in the airways. However, there is some evidence supporting the inflammatory basis of EIB, such as release of epithelial cells, mast cell mediators, and eicosanoids into the airways during EIB [38]. It has been reported that EIB-only athletes have neutrophil-dominant or mixed-type sputum while patients having EIB with asthma have eosinophil dominant inflammation in their sputum [3]. Although there have been studies on airway inflammation in EIB in athletes, the inflammatory nature of EIB in the general population has not been widely reported. In the present study, while neutrophil-dominant or mixed-type inflammations were not observed, sputum eosinophilia was commonly found in EIB patients, 57.1% of EIB-only patients and 70.6% of methacholine-hypersensitive EIB patients. This suggests a difference in the nature of inflammation between EIB-only in athletes and EIB in the general population. These results support the importance of the role of eosinophilic inflammation in the pathomechanism of EIB in the general population. Anderson et al. suggested that EIB is one of the earliest signs of chronic asthma and one of the last symptoms to disappear upon treatment with inhaled corticosteroids [39]. To clarify this suggested temporal association, a longitudinal cohort study of EIB subjects is needed.

In order to standardize testing procedures, the American Thoracic Society (ATS) recommends using the exercise provocation test under the following conditions: temperature between 20°C and 25°C and relative humidity of less than 50% [4]. When viewed in light of these criteria, there is some concern that the exercise provocation test results of this study could contain false-positive and false-negative results. However, taking into consideration the actual environments patients are exposed to, testing under the conditions proposed by the guidelines could lead to missing some patients with EIB [19]. The ATS guideline may not appropriately reflect some outdoor conditions, such as the arctic or subarctic climate regions, and winter in temperate regions. In South Korea, most males undergo compulsory military service in their early twenties and EIB occasionally develops during the winter even in subjects who have never experienced asthmatic symptoms before joining the military. Therefore, when the exercise test results are negative in persons who are suspected to have EIB alone, changing the test conditions should be considered in order to determine the presence of EIB.

In this study, we have shown that isolated EIB and EIB with asthma are heterogeneous phenotypes and test conditions, such as temperature and humidity, are critical in determining EIB positivity in isolated EIB patients. However, the pathophysiology behind the differences has yet to be elucidated, and further studies will be needed to clarify the differences.
